# Therapeutic Notes

**Published:** 1896-03-14

**Authors:** 


### THERAPEUTIC NOTES.

### CITROPHENE AND APOLYRIN—NEW ANTI-PYRITICS AND ANTI-NEURALGICS.

**Citrophene.**—This drug is a new representative of that large class of bodies which contain the para-phenetidine group, and of which phenacetine (para-aces-phenetidine) is the best known example. It contains, however, two more groups of the para-phenetidine radicle than do either phenacetine or lactophenin. Its constitution is clearly shown by the following equation:—

$$3\,C_6H_4\left\langle\begin{matrix}OC_2H_3\\NH_2\end{matrix}\right. + \begin{matrix}CH_2\quad COOH\\ |\\ C(OH).\ COOH\\ |\\ CH_2.\quad COOH\end{matrix}$$

Para-phenetidine + Citric acid

$$= C_3H_4OH\left(\begin{matrix}CONH\\OC_2H_5\end{matrix}\right\rangle C_6H_4\Big)^3 + 3\,H_{20}$$

Citrophene. Water.

Citrophene is a white powder with an odour something like citric acid, with a refreshing after taste, soluble in about four parts of cold water. It is decomposed into its constituent parts by either alkalis or acids. From its analogy to phenacetine it was regarded as perfectly safe drug for experimentation on the human subject, and from a series of experiments on typhoid and phthisical patients this was found to be the case. In doses of 5-15 grains it was found that the temperature was materially reduced, and the condition of the patient rendered more comfortable. Although in England neither of these conditions is usually treated by the exhibition of antipyretics of this kind, it is a useful indication of the action of this new drug. In cases of migraine and neuralgia, citrophene has been found useful in the hands of Benario,[1] and in this association it is said to be more active than phenacetine, owing to the large proportion of the para-phenetidine group. It is certainly, on *à priori* grounds, a drug which should recommend itself to the notice of the profession in England.

**Apolyrin** is a very similar body to the foregoing. It is described by Neucki and Jaworski[2] as being different from phenacetine, in that one atom of H. in the amide group present in para-phenetidine is replaced by the radicle of citric acid. If this is the case chemically it must be very closely associated with citrophene. It appears, however, to be less soluble (1 in 28) in cold water. It is eliminated in the urine as para-amido phenol and para-phenetidine. It is a powerful anti-pyretic and anti-neuralgic, and is said to act more promptly than similar drugs, but is contra-indicated in fasting conditions and gastric hyperacidity.

[1] Benario, D.Med., Wefnscrit, No. 26, 1895. [2] Press Méd., Oct. 26, 1895.

